# Exercise Management for Young People With Type 1 Diabetes: A Structured Approach to the Exercise Consultation

**DOI:** 10.3389/fendo.2019.00326

**Published:** 2019-06-14

**Authors:** Tarini Chetty, Vinutha Shetty, Paul Albert Fournier, Peter Adolfsson, Timothy William Jones, Elizabeth Ann Davis

**Affiliations:** ^1^Children's Diabetes Centre, Perth Children's Hospital, Perth, WA, Australia; ^2^UWA Centre for Child Health Research, University of Western Australia, Perth, WA, Australia; ^3^School of Human Sciences, University of Western Australia, Perth, WA, Australia; ^4^Telethon Kids Institute, Perth Children's Hospital, Perth, WA, Australia; ^5^Department of Pediatrics, The Hospital of Halland, Kungsbacka, Sweden; ^6^Institute of Clinical Sciences, Sahlgrenska Academy at University of Gothenburg, Gothenburg, Sweden

**Keywords:** Type 1 diabetes, exercise, physical activity, hypoglycemia, blood glucose, child, adolescent, consultation

## Abstract

Regular physical activity during childhood is important for optimal physical and psychological development. For individuals with Type 1 Diabetes (T1D), physical activity offers many health benefits including improved glycemic control, cardiovascular function, blood lipid profiles, and psychological well-being. Despite these benefits, many young people with T1D do not meet physical activity recommendations. Barriers to engaging in a physically active lifestyle include fear of hypoglycemia, as well as insufficient knowledge in managing diabetes around exercise in both individuals and health care professionals. Diabetes and exercise management is complex, and many factors can influence an individual's glycemic response to exercise including exercise related factors (such as type, intensity and duration of the activity) and person specific factors (amount of insulin on board, person's stress/anxiety and fitness levels). International guidelines provide recommendations for clinical practice, however a gap remains in how to apply these guidelines to a pediatric exercise consultation. Consequently, it can be challenging for health care practitioners to advise young people with T1D how to approach exercise management in a busy clinic setting. This review provides a structured approach to the child/adolescent exercise consultation, based on a framework of questions, to assist the health care professional in formulating person-specific exercise management plans for young people with T1D.

## Introduction

Regular physical activity during childhood is essential to promote optimal physical (1, 2) and psychological (3, 4) development. Physical activity is a key part of childhood and is not limited to sport and other forms of structured exercise; it encompasses playing and being generally physically active. However, it is well-recognized that many families do not adopt the recommendations that children and adolescents should engage in 60 min or more of physical activity daily (5). Many factors contribute to the fact that a majority of young people do not achieve recommended time spent in activity, including perceived lack of time, motivation and resources. In addition to these factors, children and young people with T1D must overcome specific challenges related to managing diabetes and exercise. These challenges include maintaining stable blood glucose levels before, during and after exercise (6) and fear of hypoglycemia (7), especially on the nights after exercise (8). Health professionals can play a key role in assisting families to overcome these challenges as physical activity confers many benefits for individuals with T1D including; improved glycemic control (9, 10), cardiovascular function (11), blood lipid profiles (10) and psychological well-being (3).

Patterns of physical activity in young people differ from adults and therefore they merit a different management approach. In young children, physical activity is usually unplanned, based- around play and often varies from day to day (12). Thus, in this young age group it can be difficult to make planned adjustments of insulin or carbohydrate, and caregivers need to be equipped to problem solve as challenges arise. In contrast, older children and adolescents may be engaging in more structured exercise such as school sports and extra-curricular activities which may involve competition (13, 14). This planned exercise provides an opportunity for sequential review and refinement of exercise strategies. In addition to changes in patterns and types of exercise during childhood, physiological responses such as changes in insulin sensitivity with growth and pubertal development impact on glucose levels (15). Furthermore, responsibilities for diabetes management change over time, with a transfer of responsibility from parents and caregivers to the increasingly independent young person.

Exercise management for young people with T1D is complex and one approach does not fit all. Many factors influence an individual's glycemic response to exercise including the type, intensity and duration of the activity (16, 17), the amount of insulin on board (18) and the person's stress/anxiety levels (19). To further complicate management, even when all these factors are kept constant, an individual's response to exercise may (20, 21) or may not be predictable on repeated exercise occasions (22).

Diabetes should not prevent individuals from achieving their exercise goals whether these are occasional fun activities or at a more high-performance level. Indeed, many individuals with T1D have gone on to accomplish extraordinary sporting achievements (23). Due to the complexity of the many factors affecting blood glucose levels, individuals often embrace a trial and error approach to manage their blood glucose levels. This approach may be compounded by the fact that health care professionals can lack the knowledge and skills or simply don't allocate time to address these challenges in a busy clinic setting. However, challenges can be overcome with appropriate training. This has been recognized most recently through the work of the JDRF sponsored Performance in Exercise and Knowledge (PEAK) initiative (24), but this excellent source of information is not pediatric specific.

There are established international pediatric exercise guidelines for the pediatric population (25) and a comprehensive pediatric-specific review of exercise in both T1D and Type 2 diabetes by Pivovarov et al. provides current perspectives and a decision tree-based approach for blood glucose management in children with T1D (26). However, it should be noted that significant gaps in the literature exist and further research is required to address these gaps. Collaborating with patients and their families at all stages of the research process should be considered when designing studies as this approach may help uptake of findings into clinical practice (27).

Although existing pediatric and general based guidelines comprehensively review the literature around diabetes management and exercise, and make recommendations for clinical practice, a gap in the literature remains in facilitating the healthcare professional to apply these evidenced- based recommendations to an exercise consultation.

The aim of this paper is to provide a structured approach for the health care professional to use in a child/adolescent exercise consultation. The goal of this approach is to facilitate the formulation of a person-specific exercise management plan. The key to this approach is ensuring recommendations are individualized and dynamic- with the use of feedback plans that are revisited and refined. A brief review of physiology is followed by the strategies or “exercise tools” commonly used in clinical practice. A series of targeted questions to structure the exercise consultation are then presented to enable selection of the appropriate exercise tool/s for the individual young person with T1D.

## Physiology

The success of the healthcare professional in assisting patients with effective exercise plans is likely to be improved by the understanding of some of the basic physiological principles of exercise and diabetes. The physiology of exercise in T1D has been reviewed in depth elsewhere (24, 28).

**Figure 1:**
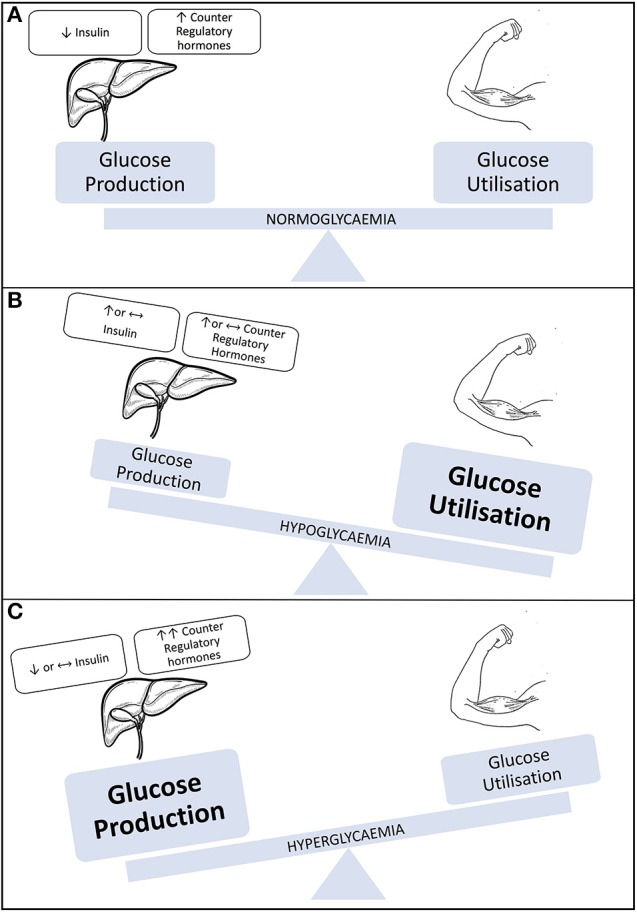
Illustration representing blood glucose levels during exercise. **(A)** Individual without type 1 diabetes/well-managed T1D. **(B)** Individual with T1D engaging in sustained aerobic activity. **(C)** Individual with T1D engaging in aerobic activity. Modified with permission from Chu et al. (31).

In an individual without diabetes, glucose provision for exercise originates predominately from the liver as a result of increased levels of glucagon and reduced circulating levels of insulin. However, during exercise in insulin-treated people with T1D, the insulin level cannot be rapidly changed, counter regulatory hormone responses can be either blunted at times or can surge as a consequence of high intensity exercise or competition (29). These hormonal imbalances can result in hypo, hyper or euglycemia (see Figure 1), which adds challenge to the management approach to exercise and diabetes (30, 31).

High individual variability exists in the blood glucose responses to different forms of exercise. In general, aerobic exercise decreases blood glucose levels, anaerobic exercise or high intensity aerobic exercise increases blood glucose levels when performed under near basal insulinemic conditions, and resistance activities are associated with relative glucose stability (32). In addition to being affected by the type, duration and intensity of the activity, individual responses are dependent on additional factors, including initial blood glucose concentrations (33), individual fitness (34), concentrations of insulin glucagon, and other counter-regulatory hormones in the circulation (35), and the nutritional status of the individual (36).

Most clinicians are familiar with the concept that insulin facilitates movement of glucose into muscle cells, but it is less commonly appreciated that muscle contraction *per se* is an insulin-independent mechanism that promotes glucose uptake into skeletal muscle with consequent additive increased risk of hypoglycemia (37). Increased glucose uptake into muscle can persist for hours after exercise and impact on post-exercise glycaemia (38).

## Exercise Tools and Management Strategies

Creating a specific exercise management plan for a young person with T1D involves understanding their pattern of physical activity, then selecting the appropriate strategy. There are a limited number of strategies available to manage blood glucose levels before, during and after exercise. These tools include glucose monitoring, carbohydrate intake, insulin adjustment, exercise strategies, and technology based tools. The choice of strategy is made easier by understanding some basic physiology of exercise and diabetes as described above.

### Glucose Monitoring

Monitoring blood glucose either by self-monitoring (SMBG) or increasingly commonly, real-time continuous glucose monitoring (rtCGM) and intermittent scanning glucose monitoring, is important for managing glycemia before, during and after exercise. Information gathered from glucose monitoring allows refinement of future exercise strategies and can inform how different factors and behaviors influence blood glucose levels. Blood glucose levels at the onset of exercise can be used to tailor glycemic management strategies.

Expert opinion suggests that although blood glucose target levels at the start of exercise should be individualized, 7–10 mmol/l is an acceptable starting range for adult patients doing aerobic exercise for up to 60-min duration (24). There are no existing guidelines for target blood glucose levels at the onset of exercise in children. However, in adults, under hyperinsulinemic conditions, the rate of fall in blood glucose levels may result in hypoglycemia if prolonged exercise is initiated within this starting range, without any adjustment to insulin dose or carbohydrate intake. Thus, in addition to starting blood glucose level, it is important to consider the rate of change of blood glucose levels to inform management decisions. Of note, although anecdotal evidence suggests a blood glucose level > 10 mmol/L may adversely affect exercise performance, studies to date have failed to demonstrate a difference in sports skill performance during acute hyperglycemia compared to normoglycemia (39, 40). Currently the evidence base is limited on whether there is an optimal blood glucose target range for exercise performance.

Continuous Glucose Monitoring (CGM) provides detailed information on not only glucose levels but trends before, during and after exercise. Information gathered from CGM can help individuals learn how different factors and behaviors influence their blood glucose levels and plan for future activities. Moreover, strategies regarding correction of hyperglycemia and prevention of hypoglycemia can be refined as information obtained from CGM could be regarded as more complete than SMBG.

What SMBG adds, as a complementary method, is an increased point accuracy as there is a lag time between blood glucose and interstitial glucose levels when glucose levels are changing rapidly, such as during exercise. This lag-time, may lead to a marked overestimation of blood glucose levels when levels are falling rapidly (41, 42). However, advancement in CGM technology has made these devices increasingly accurate (43, 44) and user-friendly. Some devices now offer the option of sharing real-time glucose levels with others or “followers”. This feature potentially allows parents/caregivers to alert their child to impending hypoglycemia during and after sports and physical activity. An observational study of 25 young people with T1D aged 8-17 years in a camp-setting showed that use of a carbohydrate intake algorithm in response to sensor glucose levels and trends prevents hypoglycemia during exercise (45).

Given that regular blood glucose monitoring provides a highly effective means to prevent hypoglycemia during and after exercise, clinicians should strongly encourage their patients not only to monitor their blood glucose levels prior to engaging in any physical activity, but also to exercise only if their glucose monitors and enough test strips are readily available or near reach (unless on CGM), particularly for situations where carrying these devices might be impractical (e.g., contact sports, swimming). Finally, clinicians should encourage their patients to measure their blood glucose levels during as well as early and for several hours after exercise, and remind them that CGM tends to overestimate blood glucose levels when they are rapidly falling.

### Carbohydrate Intake

Carbohydrate consumption before, during and after exercise can be used to prevent and treat exercise -mediated hypoglycemia (46). Factors influencing the amount of carbohydrate intake required to prevent exercise-mediated hypoglycemia include body mass, circulating insulin levels and the type, intensity and duration of exercise. The blood glucose level and trend at the start of exercise are other factors to consider and recommendations based on these parameters should be individualized.

The carbohydrate requirement to prevent exercise-mediated hypoglycemia increases with plasma insulin levels (47), with the pattern of blood glucose response to exercise being highly unpredictable under hyperinsulinaemic compared to near basal insulinaemic conditions (22).

If exercise is occurring when circulating insulin levels are high, such as within 3 h of a meal-time insulin bolus, then up to 1.0–1.5 g of carbohydrate/kg ideal body weight/ hour of sustained activity may be required (48, 49). In contrast, when exercise is taking place several hours after a meal-time bolus, or when the insulin bolus dose has been lowered pre-exercise, then carbohydrate requirements are lower (~0.3–0.5 g/kg ideal body mass/h (47, 49). When insulin levels are close to basal levels, such as when exercise is performed before breakfast, the risk of hypoglycemia is minimal (50) and carbohydrate supplementation may not be required (51, 52). Given that categorizing carbohydrate requirements in a dichotomous manner (high vs. low insulin levels) is a simplified approach that does not take into account all the factors influencing glucose requirements, Francesco et al have developed an algorithm that estimates an individuals' glucose requirement during activity based on personalized situation specific information including insulin concentration (53).

In addition to being affected by insulin levels, the amount of carbohydrate to prevent exercise mediated hypoglycemia varies with the intensity and duration of exercise (18). A study in young people with T1D exercising under basal insulin levels reported that glucose requirements to maintain euglycemia during 40 min of exercise increase with intensity up to 50% and 65% VO_2_ peak, but with no glucose required at 80% VO_2_ peak (54). It remains to be established if this pattern is still observed when exercise is performed for a longer duration or under high circulating insulin levels.

The type and timing of carbohydrate ingestion should also be considered. Carbohydrates with a high glycemic Index (GI) such as glucose in liquid, tablet, and gel forms, are digested and absorbed more quickly, resulting in a rapid rise in blood glucose levels. In contrast, low GI foods, including fruits and wholemeal bread, are released more slowly causing a gradual and sustained rise in glycemia (55, 56).

A meal or snack containing low GI carbohydrate 1–4 h prior to exercise can increase hepatic glycogen stores and provide sustained carbohydrate release during exercise (57, 58). In contrast, high GI carbohydrates are preferable immediately prior to and during prolonged exercise (59). High GI snacks are also recommended in early recovery (1–2 h post exercise) to replenish glycogen stores (60) and to avoid hypoglycemia in this period of heightened insulin sensitivity. A bedtime snack containing carbohydrate, fat and protein may help reduce the risk of hypoglycemia on nights following exercise (61).

Clinical recommendations for carbohydrate intake will vary if the goal is hypoglycemia prevention, weight reduction, improving glycaemic control or optimal exercise performance. Since high carbohydrate intake is often recommended for healthy individuals without diabetes before and during prolonged exercise to optimize endurance performance (60), this strategy has recently been explored in adults with T1D (62), The authors report that increased carbohydrate supplementation, matched with increased insulin doses, is safe and allows the prevention of hypoglycemia during prolonged aerobic activity (62). This strategy has not been explored in the pediatric population. It is important to match insulin dose with extra CHO intake as excessive carbohydrate supplementation without matched insulin may result in hyperglycemia (63).

### Insulin Adjustment

Insulin adjustment, along with balanced carbohydrate intake, is a key tool for managing blood glucose levels during and after exercise. The degree to which blood glucose levels fall during exercise is dependent on the amount of circulating insulin (47). Reduction in insulin doses to prevent exercise-mediated hypoglycemia is typically required for prolonged (>30 min) moderate intensity exercise, particularly if insulin is above basal levels (18, 64).

Options for insulin adjustment vary depending on insulin regimen. Insulin pumps allow greater flexibility in adjusting basal rates than injection regimens. Therefore, basal dose adjustments are most relevant to patients on insulin pumps, particularly for unplanned exercise, whereas bolus dose adjustment can be applied to most regimens. For those on Multiple Daily Injections (MDI), basal dose reduction can reduce the risk of nocturnal hypoglycemia on nights following afternoon exercise (25). However, this approach may result in hyperglycemia, and if this is the case, should be reserved for when participating in more activity than usual, such as sports camps.

**Table 1:** Strategies to manage blood glucose levels during and after exercise.

**Problem**	**Mechanism**	**Tool**	**Strategy**
Hypoglycemia during exercise or recovery	• During sustained aerobic activity, relative insulin excess suppresses hepatic glucose production • Mismatch between glucose production by liver and increased glucose utilization by skeletal muscles	Carbohydrate intake	AMOUNT: Less than 3 h since last insulin bolus: up to 1.0–1.5 g of carbohydrate/kg ideal body weight/h Greater than 3 h since last insulin bolus: ~0.3–0.5 g/kg ideal body mass/h TYPE: Low GI CHO 1–4 h prior to exercsie High GI CHO immediately prior, during and just after activity
		Insulin adjustment	Less than 3 h since last insulin bolus: Consider bolus dose reduction of 25–75% Greater than 3 h since last insulin bolus: basal rate reduction of 50–80% started 60–90 min prior to activity
		Exercise based strategies	10 s sprint before, during or after exercise
		Technology	CGM + carbohydrate intake algorithm SAP (LGS or PLGS) or closed loop therapy
Hypoglycemia on nights after exercise	• Relative insulin excess • Increased glucose uptake by skeletal muscles (increased insulin sensitivity) • Blunted counter regulatory response after exercise and during sleep	Carbohydrate intake	Meal after exercise to replenish glycogen stores Consider bedtime snack containing CHO, fat and protein
		Insulin adjustment	Pump: Basal rate reduction overnight (e.g., 20% reduction for 6 h) MDI: Consider basal analog reduction (e.g., 20% reduction in combination with bedtime snack)
		Technology	CGM with alarms SAP (LGS or PLGS) or closed loop therapy
Hypoglycemia during exercise or recovery	• Anaerobic activity causes increased lactate and counter regulatory hormones	Insulin adjustment	Consider conservative corrective bolus dose (e.g., 50% of usual dose)
		Exercise based strategies	Aerobic cool-down session

### Exercise-Based Strategies

Utilization of the glycemic effects of specific types of exercise can play a role in managing blood glucose levels. The blood glucose rising effect of sprinting in young people with T1D suggests that this may provide a strategy to reduce hypoglycemia risk during and after exercise (65). Indeed, a series of studies in young adults with T1D have shown that a maximal 10 s sprint performed before (66) or after (67) moderate intensity exercise can prevent blood glucose levels from falling early after exercise. Furthermore, frequent short sprints (4 s sprints every 2 min) during moderate intensity exercise reduce the decline in blood glucose levels compared to continuous moderate intensity exercise during and early after exercise (68, 69). These strategies have been trialed in clinic-based studies, but real-world data are lacking.

In contrast, exercise induced hyperglycemia can be managed with moderate intensity exercise. Anecdotal evidence in adults suggests that moderate intensity exercise can be used as a strategy to counteract exercise-induced hyperglycemia, a technique known as an “aerobic cool down” (70). The mechanisms underlying this technique remain to be well-understood, they may include increased glucose (71) and lactate oxidation. Lactate is a substrate for gluconeogenesis and would potentially otherwise be converted to glucose in the liver resulting in hyperglycemia (72, 73).

### Advances in Diabetes Technology

Diabetes technology is rapidly evolving and provides further tools to aid glucose management and reduce the burden of diabetes during exercise (74). Technology based tools include smart phone applications, insulin pumps, CGM, sensor augmented pump therapy and closed loop technology. Smart phone applications can help individuals to record exercise management and aid exercise management decisions (75). Insulin pumps enable greater flexibility in insulin adjustment as basal rates can be reduced or stopped before during or after exercise to prevent exercise mediated hypoglycemia. Despite some issues with sensor accuracy at times of rapid glucose flux, CGM can provide comprehensive dynamic real-time glucose information around exercise to help inform diabetes management decisions.

Perhaps the biggest game changer in current diabetes management is the ability to link sensor glucose readings to insulin delivery in sensor augmented and closed loop pump therapy. Sensor augmented pump (SAP) therapy includes low glucose suspend and predictive low glucose suspend technology, where a mathematical algorithm suspends basal insulin delivery when sensor glucose levels fall below, or are predicted to fall below a pre-set threshold. Studies investigating the use of low glucose suspend and predictive low glucose suspend technology during exercise have shown a reduction in hypoglycemia (21, 76).

Closed loop systems expand on the concept of SAP therapy by using a control algorithm that continually increases or decreases hormone delivery in response to sensor glucose levels. Systems include dual hormone (insulin and glucagon) and single hormone (insulin only) pumps. Closed loop technology may be seen as the future of diabetes management, however physical activity remains one of the biggest challenges to fully automated systems (77). This is because physical activity in individuals with T1D induces rapid changes in glucose levels due to hormone imbalances that are dependent on the type, duration and intensity of the activity as well as individual factors such as fitness levels and the amount of circulating insulin on board. Furthermore, the glucose trends during physical activity may be variable within and between individuals. Further research is needed to guide exercise recommendations in patients using the current closed loop systems

For closed loop systems to be effective at this time of rapid and potentially unpredictable glucose fluctuations, several challenges must be overcome including sensor glucose lag-time (as previously discussed), pharmacokinetics of subcutaneous insulin administration and how to sense not only that physical activity is occurring but the intensity of the activity taking place (77).

In an individual without T1D, insulin levels rapidly decrease at the onset or exercise. This is difficult to replicate in T1D as insulin is administered subcutaneously and levels are not under endogenous control. Furthermore, absorption of insulin may be increased from stores of already administered subcutaneous insulin during exercise due to increased blood flow to the working muscle (78). Bihormonal pumps infusing both glucagon and insulin may help overcome this challenge (79).

Closed loop systems may be better able to manage blood glucose levels during exercise if the system could “sense” the intensity and duration of the physical activity. Future developments may include integration of physical activity monitoring such as heart rate and or accelerometery data into closed loop systems.

## A Structured Series of Questions for the Exercise Consultation

**Figure 2:**
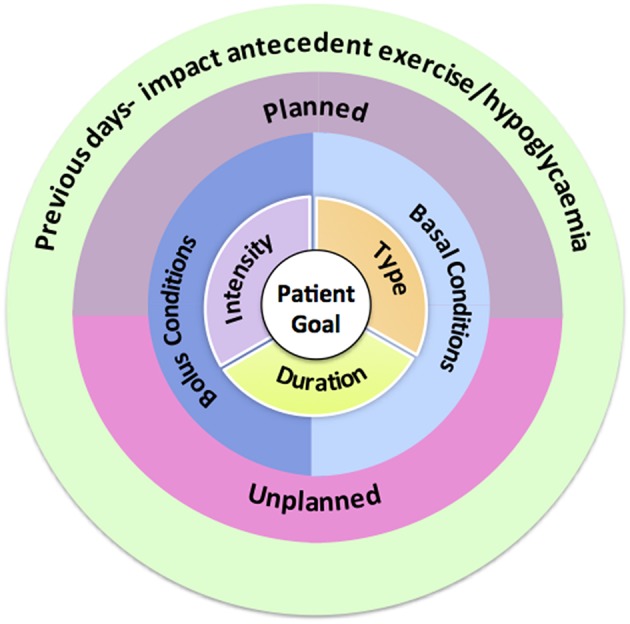
Structured approach to the exercise consultation.

A structured approach to exercise management in young people with Type 1 diabetes involving targeted and clinically relevant questions can provide the framework for an effective consultation and aid selection of the most appropriate tools as described above. We recommend starting from the center of Figure 2 and working outwards. Some health care professionals may find the use of a consistent template that addresses each of the 5 questions helpful. These questions can be re-visited in sequential consultations together with information from glucose monitoring to refine recommendations.

### Q1. What Are the Individual's Exercise and Glycemic Goals?

The first step in formulating a patient's exercise management plan involves identifying the patient's exercise goal, as this will influence subsequent management decisions. Exercise goals may include: weight loss, exercise performance, socialization and fitness. Glycemic goals in addition to hypoglycemia prevention may include, maintaining blood glucose levels in target range, or achieving a specific blood glucose level to maximize exercise performance.

If the exercise goal is facilitating weight loss, it would be most appropriate to choose an insulin-reduction based strategy to manage blood glucose level during and after exercise so as to avoid excessive CHO intake particularly after exercise. If socialization and fun is the goal, then insulin dose reduction may be key to minimize time spent out of the activity to test blood glucose level and ingest CHO. In contrast, if the primary goal is exercise performance, then an individual may be aiming for increasing CHO intake before (e.g., CHO loading) and during exercise to maximize performance while managing their blood glucose levels so as to minimize or avoid any interruptions.

Strategies for exercise performance may focus on matching carbohydrate intake to the specific nutritional requirement of the activity (rather than hypoglycemia prevention) and insulin doses should be increased accordingly (62). If fitness is the goal, and the patient has a plan to incrementally increase physical activity over time, it will be important to anticipate a reduction in total daily insulin requirement to avoid hypoglycemia as insulin sensitivity increases in response to exercise training.

### Q2. What Is the Exercise Type, Duration and Intensity?

The next step is to consider, the type, duration and intensity of the physical activity. This will allow anticipation of the expected effect on glucose levels (24). As mentioned above, continuous aerobic activity performed when insulin levels are high will result in a decrease in blood glucose levels and require a reduction in insulin and/or an increase in carbohydrate supplementation to avoid hypoglycemia. In contrast, short duration high intensity aerobic or anaerobic activity, a pattern typical of children's natural play (80), may maintain glucose levels during exercise and even result in early post-exercise hyperglycemia (16, 81) if performed while plasma insulin is close to basal level. Competition-induced anticipatory stress may also raise blood glucose levels as a result of elevated catecholamine levels.

Exercise-related hyperglycemia may require a conservative insulin correction dose (50% of usual dose) administered immediately after exercise (82). Alternatively, an aerobic cool-down session can be used to counteract post-exercise hyperglycemia (70). Since such a use of insulin or exercise as a means to oppose hyperglycemia post-exercise may increase the risk of hypoglycemia if the insulin dose or the duration of the cool-down session is excessive these strategies should be adopted only if accompanied by regular blood glucose monitoring.

### Q3. Timing of Exercise Relative to the Last Insulin Bolus Dose?

Establishing the timing of exercise in relation to the last insulin bolus provides insight on relative insulin levels and will guide decisions on insulin adjustment, in particular if reductions should be made to basal or bolus insulin doses.

When exercise is planned to take place within 3 h of administration of an insulin bolus (high insulin levels), then a bolus dose reduction of 25-75% can be trialed (18, 83) It should be noted that the smaller the insulin dose administered, the shorter its duration of action (84). Recommendations for bolus reduction should be relative to the duration and intensity of the exercise (24). Alternatively, or in combination with insulin adjustment, pre-exercise carbohydrate intake to up to 1.0–1.5 g of carbohydrate/kg ideal body weight/ hour of sustained activity (85) may be required as discussed above.

Exercise performed when plasma insulin is close to basal levels (e.g., in the morning prior to breakfast) is less likely to result in hypoglycemia as circulating insulin levels are typically low. If hypoglycemia is occurring in this setting then management options include: carbohydrate consumption or reduction of basal insulin. Carbohydrate intake to prevent hypoglycemia when insulin levels are low is approximately 0.3-0.5 g/kg ideal body mass/hr; (47, 49). Reduction of basal insulin is more easily achieved for individuals on pump regimens and recommendations include a temporary basal rate reduction of 50-80% (64). To be effective, basal rates should ideally be reduced 60–90 min prior to the onset of activity. Other strategies to consider include use of automated basal insulin suspension functions during and after exercise, including: low glucose suspend (76) and predictive low glucose suspend technology (21).

Late onset post exercise hypoglycemia is the phenomenon of overnight hypoglycemia particularly occurring after late afternoon exercise (38, 86). A basal reduction; for those on insulin pumps a basal rate reduction of 20% for 6 h (87), or for those on MDI a 20% basal dose reduction in combination with a carbohydrate snack at bedtime (25), can reduce the incidence of nocturnal hypoglycemia in response to a prior bout of afternoon exercise.

### Q4. Is the Exercise Planned or Spontaneous?

It is helpful to ascertain if the individual engages in mostly planned or spontaneous physical activity. If exercise is planned, then strategies based on insulin adjustment with or without carbohydrate supplementation can be implemented (see above). Furthermore, exploring if planned events involve the same type of activity at the same time of day will help establish if glycemic patterns are reproducible for that individual on different occasions when conditions are similar. If exercise is spontaneous and pre-exercise adjustment of bolus and basal insulin are not available in advance, then the options available are limited to basal insulin reduction, carbohydrate supplementation or use of glycemia-rising high intensity exercise (67, 81).

### Q5. Have There Been any Episodes of Hypoglycemia and /or Exercise Prior to the Exercise Session?

When reviewing the glycemic response to a period of exercise it is helpful to find out if there have been any episodes of hypoglycemia or exercise prior to the exercise event. Both preceding exercise (88) and hypoglycemia (89) can attenuate the counter-regulatory response to subsequent exercise thereby increasing the risk of hypoglycemia.

The pattern and frequency of prior exercise is informative. Regular participation in aerobic activity increases insulin sensitivity and may consequently lower the total daily insulin requirement (90, 91). Therefore, if exercise is occurring on a day to day basis, further insulin dose reduction may be required.

## Conclusion

It can be challenging for health care practitioners to advise young people with T1D how to approach exercise safely. Clinical guidelines and a recent comprehensive consensus statement on exercise and T1D provide thorough evidence-based recommendations. This paper aims to facilitate the healthcare professional in applying such evidence-based recommendations to clinical practice using a structured approach to the exercise consultation based on a framework of relevant questions. The tools or management strategies are provided as a starting base to help clinicians work with patients and families to achieve their exercise goals. This framework of questions together with information from blood glucose monitoring should be re-visited at sequential consultations to allow refinement of future exercise management strategies.

## Author Contributions

TC was responsible for the manuscript preparation. VS, PF, PA, TJ, and ED reviewed and edited the manuscript. All authors approved the final version of this article.

### Conflict of Interest Statement

The authors declare that the research was conducted in the absence of any commercial or financial relationships that could be construed as a potential conflict of interest.

## Abbreviations

T1D: Type 1 Diabetes
SMBG: Self-monitoring blood glucose
CGM: Continuous glucose monitoring
rtCGM: Real-time continuous glucose monitoring
SAP: Sensor Augmented Pump.
